# Ablation of p75^NTR^ signaling strengthens gamma–theta rhythm interaction and counteracts Aβ-induced degradation of neuronal dynamics in mouse hippocampus in vitro

**DOI:** 10.1038/s41398-021-01332-8

**Published:** 2021-04-09

**Authors:** Yuniesky Andrade-Talavera, Hugo Balleza-Tapia, Pablo Dolz-Gaitón, Gefei Chen, Jan Johansson, André Fisahn

**Affiliations:** 1 Neuronal Oscillations Laboratory, Division of Neurogeriatrics, Department of Neurobiology, Care Sciences and Society, Center for Alzheimer Research, Karolinska Institutet, 17164 Solna, Sweden; 2 Division of Neurogeriatrics, Department of Neurobiology, Care Sciences and Society, Center for Alzheimer Research, Karolinska Institutet, 14183 Huddinge, Sweden

**Keywords:** Diseases, Physiology

## Abstract

Gamma and theta brain rhythms play important roles in cognition and their interaction can affect gamma oscillation features. Hippocampal theta oscillations depend on cholinergic and GABAergic input from the medial septum-diagonal band of Broca. These projecting neurons undergo degeneration during aging and maintain high levels of neurotrophin receptor p75 (p75^NTR^). p75^NTR^ mediates both apoptosis and survival and its expression is increased in Alzheimer’s disease (AD) patients. Here, we investigate the importance of p75^NTR^ for the cholinergic input to the hippocampus. Performing extracellular recordings in brain slices from p75^NTR^ knockout mice (p75^−/−^) in presence of the muscarinic agonist carbachol, we find that gamma oscillation power and rhythmicity are increased compared to wild-type (WT) mice. Furthermore, gamma activity is more phase-locked to the underlying theta rhythm, which renders a stronger coupling of both rhythms. On the cellular level, we find that fast-spiking interneurons (FSNs) fire more synchronized to a preferred gamma phase in p75^−/−^ mice. The excitatory input onto FSN is more rhythmic displaying a higher similarity with the concomitant gamma rhythm. Notably, the ablation of p75^NTR^ counteracts the Aβ-induced degradation of gamma oscillations and its nesting within the underlying theta rhythm. Our results show that the lack of p75^NTR^ signaling could promote stronger cholinergic modulation of the hippocampal gamma rhythm, suggesting an involvement of p75^NTR^ in the downregulation of cognition-relevant hippocampal network dynamics in pathologies. Moreover, functional data provided here suggest p75^NTR^ as a suitable target in the search for efficacious treatments to counteract the loss of cognitive function observed in amyloid-driven pathologies such as AD.

## Introduction

The synchronization of many of the different interconnected neuronal assemblies present in the hippocampal microcircuitry gives rise to the emergence of what is known as neuronal oscillations^1^. From the several oscillatory rhythms identified in the brain, two are intimately associated with cognitive tasks linked to the hippocampus, that is, learning and memory. These are the gamma (30–80 Hz) and theta (5–10 Hz) rhythms^2^, which can occur separately, but are also known to coexist and interact^3^. The resulting *nesting* of gamma oscillations within, or on top of, theta oscillations, also known as cross-frequency coupling, can greatly affect the characteristics of the gamma rhythm. Several interactions have been reported allowing a hierarchical organization of the rhythms that leads to precise neuronal firing patterns. The most common interaction that has been reported is the coupling between theta phase and gamma power^4–10^. Theta-phase to gamma-phase^11,12^ and theta power to gamma power couplings have also been described^12^.

Some physiological advantages of theta–gamma coupling are the possibility of encoding different information in different theta phases and the synchronization of neuronal ensembles over long distances^13,14^. It is noteworthy that in addition to modulating gamma oscillations, the theta rhythm can also induce them^15^.

Hippocampal theta rhythm depends partially on the cholinergic and GABAergic input from the medial septum and diagonal band of Broca (MS/DBB) located in the basal forebrain, which has been classically considered as the “pacemaker” for the hippocampal theta activity^16^. Cholinergic fibers are known to innervate both pyramidal cells and interneurons in the hippocampus. Selective septo-hippocampal cholinergic lesions sparing GABAergic inputs can affect the amplitude of theta oscillations^17^. Thus, any factors that influence the development and/or survival of basal forebrain cholinergic neurons (BFCNs) are likely to have a profound impact on the dynamics of cognition-relevant oscillatory behavior in the hippocampus.

Cholinergic neurons of the MS/DBB are known to undergo a moderate degeneration during aging resulting in a decrease of cholinergic function that has been related to the progressive memory deficits during aging^18,19^. This age-dependent degeneration of BFCN might be due to a decrease in trophic support, as in aging an attenuation of the neurotrophic signaling in sensory neurons has been observed^20^. In hippocampal slices, it has been observed that selective ablation of the neurotrophin receptor TrkB in fast-spiking interneurons (FSNs) decreases the amplitude of carbachol (CCh)-induced gamma oscillations^21^. However, much less is known about how neurotrophic signaling may regulate neuronal network rhythms and paramount neuronal populations such as FSNs.

On the other hand, neurotrophins are also known to affect the viability, differentiation, and size of cholinergic neurons^22–24^. The neurotrophin receptor p75 (p75^NTR^) has been reported to mediate both proapoptotic signaling and survival, as well as neurite growth^25^. BFCNs are among the few neuronal cell types that maintain p75^NTR^ expression at high levels during adulthood. Van der Zee et al.^26^ first reported an increased number of cholinergic neurons in the septum of mice carrying a targeted mutation in the third exon of the *p75*^*NTR*^ gene. Conversely, p75^NTR^ overexpression significantly reduces dendritic complexity as well as spine density in CA1 pyramidal cells^27^. In addition, p75^NTR^ interacts directly with amyloid-beta peptide (Aβ) mediating neuritic dystrophy^28,29^.

Such interaction points toward the deleterious effect of the presence of p75^NTR^ in age-related neurodegenerative diseases like Alzheimer’s disease (AD). Although p75^NTR^ has an evident effect on the overall cholinergic function in the brain, particularly on the survival and innervation of BFCN having the potential to modulate both hippocampal theta and gamma oscillations, the role of p75^NTR^ on these oscillatory rhythms in normal and pathological conditions remains unknown. As such, it has been observed that Aβ induces dysfunction of glutamatergic neurons impairing septum rhythmicity. This may negatively affect hippocampal rhythmogenesis and underlie the memory loss observed in AD^30^.

Here, we describe the effect of p75^NTR^ ablation of increasing cognition-relevant hippocampal rhythms such as gamma oscillations, elucidate the underlying cellular mechanisms in FSN, and how it strengthens the interaction with the underlying theta oscillations in mouse brain slices. We show that lack of p75^NTR^ signaling protects the hippocampal network against pathophysiological changes observed in AD such as the Aβ-induced degradation of gamma oscillations and gamma–theta interaction. It suggests p75^NTR^ signaling inhibition as a potential focus for novel therapies against AD including cognitive-relevant rhythms strengthening.

## Methods

### Animals

Experiments were performed in accordance with the ethical permit granted by Norra Stockholms Djurförsöksetiska Nämnd to AF (N45/13). p75^*ExonIII+/−*^ mice (p75^+/−^) with a C57BL/6J background were purchased from Jackson Laboratory (USA). Animals were inbred in order to obtain the p75^+/+^ and p75^−/−^ mice used for experiments. The number of animals was reduced to a minimum by optimizing the use of slices from both hemispheres. The total number of animals was set to a minimum of 3 and a maximum of 5 per group and condition. Male animals between P15 and P21 were deeply anesthetized using isofluorane before being sacrificed by decapitation and brain removal.

### Genotyping

Genotypic analysis was performed by PCR using primer *p75com* (5′-GGACAAACAGAACACAGTGTGTGA-3′) in exon III upstream of the insertion site, and primer *p75wt* (5′-ACCCATATAATCGCTGAGAGAGGA-3′) in the following intron for the detection of the wild-type allele (437 bp amplicon, Fig. S1) and primer *p75ko* (5′-GAACTTCCTGACTAGGGGAGGAGT-3′) in the PGK promoter of the vector inserted^26^ for the detection of knockout allele (252 bp amplicon, Fig. S1). Cycle parameters were 94 °C for 4 min, 95 °C for 20 s, 60 °C for 20 s, 72 °C for 32 s, 40 cycles, and 72 °C for 7 min.

### Hippocampal slice preparation

Hippocampal slices were prepared as previously described^31^. Briefly, the brain was dissected out and placed in ice-cold artificial cerebrospinal fluid (ACSF) modified for dissection containing (in mM) 80 NaCl, 24 NaHCO_3_, 25 glucose, 1.25 NaH_2_PO_4_, 1 ascorbic acid, 3 Na pyruvate, 2.5 KCl, 4 MgCl_2_, 0.5 CaCl_2_, 75 sucrose, and bubbled with carbogen (95% O_2_ and 5% CO2). Horizontal sections (350 µm thick) of the ventral hippocampi of both hemispheres were prepared with a Leica VT1200S vibratome (Leica Microsystems). After cutting, slices were transferred into a humidified interface holding chamber containing standard ACSF (in mM): 124 NaCl, 30 NaHCO_3_, 10 glucose, 1.25 NaH_2_PO_4_, 3.5 KCl, 1.5 MgCl_2_, and 1.5 CaCl_2_, continuously supplied with humidified carbogen gas (5% CO_2_, 95% O_2_). The chamber was held at 37 °C during slicing and subsequently allowed to cool down to room temperature for at least 1 h before the commencement of experiments.

### Electrophysiology

For local field potential (LFP) recordings glass microelectrodes (4–6 MΩ) filled with standard ACSF were placed in CA3 stratum pyramidale. Single-cell recordings were carried out in a submerged recording chamber. Action potentials (APs) and excitatory postsynaptic potentials (EPSCs) from FSNs in area CA3 were recorded in whole-cell patch-clamp mode with an internal recording solution containing (in mM): 122.5 K^+^ gluconate, 8 KCl, 4 Mg^2+^ATP, 0.3 Na^+^GTP, 10 HEPES, 0.2 EGTA, 2 MgCl_2_ with pH set to 7.2–7.3, and osmolarity to 270–280 mosmol/l. AP firing was recorded in current clamp at free membrane potential.

FSNs were visualized under an upright microscope using infrared-differential interference contrast (IR-DIC) microscopy (Axioskop, Carl Zeis AG, Göttingen, Germany) and were identified and distinguished from other interneuron populations based on their morphology and characteristic firing behavior (Fig. S2)^32,33^ and location in the CA3 area. Interface chamber LFP recordings were performed with a 4-channel M102 amplifier (University of Cologne, Germany). Data were sampled at 5 kHz, conditioned using a HumBug 50 Hz noise eliminator (Quest Scientific), low-pass filtered at 1 kHz, digitized (Digidata 1440A, Molecular Devices, CA, USA), and stored on a hard drive using pCLAMP 9.6 software (Molecular Devices). Concomitant LFP and patch-clamp recordings in the submerged-type recording chamber were performed with a patch-clamp amplifier (Multiclamp 700B), and data were acquired using pCLAMP 10.4 software (Molecular Devices). LFP recordings were also conditioned using a HumBug 50 Hz noise eliminator (Quest Scientific). All signals recorded in submerged configuration were low-pass filtered at 1 kHz, acquired at 5 kHz, and digitized and stored using Digidata 1322A and pCLAMP 10.4 software (Molecular Devices, CA, USA).

In all experiments, gamma oscillations were elicited by applying CCh (20 μM) to the extracellular bath^34^. Oscillations were allowed to stabilize for at least 20 min before recording.

### Data analysis

Fast Fourier transformations for power spectra were calculated from 60-s-long LFP data traces (segments of 8192 points) using Axograph X software (Kagi, Berkeley, CA, USA). Gamma oscillation power was calculated by integrating the power spectral density from 20 to 80 Hz using KaleidaGraph. Theta oscillation power was calculated on 4–10 Hz filtered traces. Gamma envelope (*γ*_ENV_, see below) power was calculated as the integrated power between 5 and 10 Hz. Peak frequency for each signal was obtained as the frequency at which the maximum power of the respective frequency band was located. Once differences in the peak frequency were detected, further confirmation was derived from analyzing the lag to the second peak in the corresponding autocorrelogram (see below). For further analysis of LFP recordings, the signals were preprocessed using a band-pass Butterworth filter (in both directions to prevent a shift in the phase angle) set to 20–60 Hz for autocorrelation analysis of gamma oscillations and 4–10 Hz (theta) for gamma and theta interaction. Normalized autocorrelations were performed using Matlab custom-written routines. The coefficient of rhythmicity (Cr) was calculated from the autocorrelograms as a measure of the quality of gamma and theta oscillations. It was defined as Cr = (*α* − *β*)/(*α* + *β*) including a modification to Andersson et al.^35^: prior to Cr calculation, (1 + *α*) and (1 + *β*) corrections were applied with *α* corresponding to the value of the height of the second peak and *β* to the first trough in the autocorrelogram (counting the first peak at zero lag). Cr ranges between 0 and 1 with higher coefficient values denoting more rhythmic activity. Only recordings having a Cr ≥ 0.01 were considered rhythmic and included in the study^36^.

A gamma amplitude time series were obtained using a Matlab custom-written routine, where the built-in envelope function (*γ*_ENV_) was set to “peak” mode and peak separation was set to 100 in order to finely adjust the envelope to the amplitude of the signal. In order to assess the reliability of the method, we have analyzed ten recordings from the control group (p75^+/+^) taken at random using different peak adjustment settings (Fig. S3). For the time domain (cross-correlation (XC)) analysis, the filtered theta oscillations (*θ*) and the *γ*_ENV_ were used. Normalized XC between *θ* and the *γ*_ENV_ (XC(*θ*,*γ*_ENV_)) was performed with a Matlab custom-written routine. The oscillatory nature of the signals was evidenced in the XC analysis that displayed several peaks with periodic fluctuations. To describe the similarity and phase shift of both signals, we considered the magnitude and lag of the central negative peak, respectively^37^.

Spike-phase coupling analysis was performed on concomitant LFP recordings and single-cell recordings using a custom-made routine in Matlab to relate the FSN spiking activity to ongoing gamma oscillations^32,33,36^. The 20–40 Hz filtered LFP traces were used and APs were detected using an amplitude threshold. The instantaneous phase angle of gamma oscillations at which an AP occurred was determined by using a Hilbert transform. Phase angles of all AP and gamma oscillation phases were represented in polar plots and expressed in radians with the peak of the oscillation cycle corresponding to 0*π* and the trough to ±*π* in the polar plots. Each AP was assigned a vector of length 1 with an angle corresponding to the phase of the field at the same time. An averaged resultant phase-density vector was used to describe the preferred phase of firing (phase angle) and how recurrent the firing in that angle was (vector length). Thus, a larger vector denotes a more synchronized AP firing. The vector length is shown normalized by the total number of AP for each cell recorded from both p75^+/+^ and p75^−/−^. All distributions were tested for uniformity. Inclusion criteria were set at *p* < 0.05 value by performing a Rayleigh’s test.

### Drugs and chemicals

All chemical compounds used in intracellular and extracellular solutions were obtained from Sigma-Aldrich Sweden AB (Stockholm, Sweden). CCh was dissolved in milliQ water.

Met-Aβ residues 1–42 (referred to as Aβ herein) were produced as previously described^38^. Briefly, Aβ was expressed in BL21*(DE3) pLysS *Escherichia coli* and purified with DEAE-Sepharose (GE Healthcare). To get rid of large aggregates, the peptides were passed through a 30,000 Da Vivaspin concentrator (GE Healthcare) at 4 °C. The filtrate was concentrated at 4 °C with a 5000 Da Vivaspin concentrator (GE Healthcare) until Aβ concentration was ~50 µM. The peptides were aliquoted in low-bind Eppendorf tubes (Axygen) and stored at −20 °C.

### Statistical analysis

All statistical analysis was performed using GraphPad Prism. Results are reported as mean ± SEM. Prior statistical analysis all the data were subjected to an outliers determination and removal with the ROUT (robust regression and outlier removal) method, followed by tests for normality distribution and variance similarity between groups. Due to previous experience with outliers and overall sample behavior, sample size was determined based on previous studies performed in interface-type chambers^31,36,38–40^ as well as in submerged-type chambers^31,33,36^. Tests for statistical significance were performed on absolute values in all the experiments using two- or one-sided when appropriate. Student’s *t* test or Mann–Whitney *U* test were performed depending on the parametric or nonparametric distribution, respectively. Significance levels were set as follows: *p < 0.05, **p < 0.01, ***p < 0.005, and ****p < 0.0001.

## Results

### Ablation of p75^NTR^ signaling increases gamma oscillation power and rhythmicity as well as gamma–theta phase locking in the hippocampal network

**Fig. 1:**
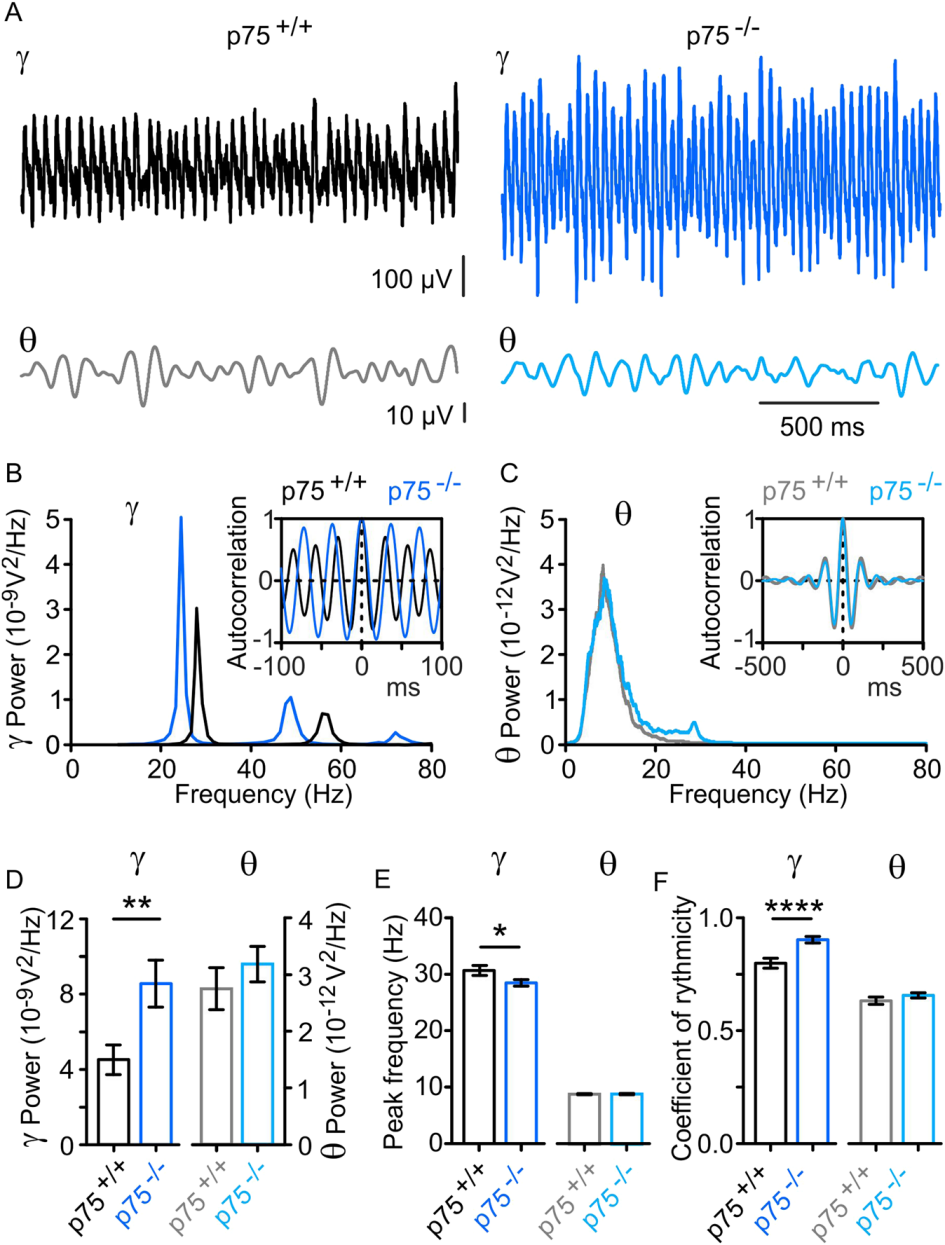
p75^NTR^ ablation induces alterations of spectral features and rhythmicity of gamma oscillations, while theta parameters remain unaltered. **A** Representative sample traces of CCh-induced gamma oscillations (*γ*, top traces) and the 4–10 Hz filtered theta component (*θ*, bottom traces) in hippocampal slices from p75^+/+^ (black) and p75^−/−^ (blue) mice, respectively, 20 min after CCh bath application. **B**, **C** Power spectra of *γ* and *θ* oscillations recorded in the experimental conditions described in (**A**). Insets: Corresponding *γ* and *θ* oscillation autocorrelograms showing a significant increase of the lag to peak measured on the second peak in p75^−/−^ mice (p75^−/−^ mice: 34.7 ± 0.7 ms, *n* = 3; p75^+/+^ mice: 31.0 ± 0.7 ms, *n* = 24, *p* = 0.0004 Student’s *t* test). **D** Mean integrated power of *γ* oscillations (20–80 Hz; p75^−/−^: 8.6 ± 1.3 × 10^−9^ V^2^, *n* = 31; p75^+/+^: 4.5 ± 0.78 × 10^−9^ V^2^, *n* = 24, *p* = 0.0067) and *θ* (4–10 Hz; p75^+/+^: 2.7 ± 0.37 × 10^−12^ V^2^, *n* = 24; p75^−/−^: 3.2 ± 0.31 × 10^−12^ V^2^, *n* = 31, *p* = 0.1856) from experiments described in (**A**), showing a significant increase in the 20–80 Hz frequency band in p75^−/−^ mice (blue), while *θ* spectrum remains unaltered. **E** Peak frequency of *γ* and *θ* spectra. *Γ* oscillations from p75^−/−^ mice appear to be slower than those recorded in p75^+/+^ mice (p75^+/+^: 30.9 ± 0.9 Hz, *n* = 24 vs. p75^−/−^: 28.7 ± 0.6 Hz, *n* = 31, *p* = 0.0175). Peak frequency of *θ* spectra remains similar in both mice strains (p75^+/+^: 8.9 ± 0.1 Hz, *n* = 24; p75^−/−^: 8.9 ± 0.1 Hz, *n* = 31, *p* = 0.2596). **F** Coefficient of rhythmicity (Cr) calculated from the autocorrelogram (see “Methods”) of *γ* oscillations reveals a higher rhythmicity of *γ* oscillations recorded in p75^−/−^ mice (p75^+/+^: 0.8 ± 0.02, *n* = 24 vs. p75^−/−^: 0.91 ± 0.01, *n* = 31, *p* < 0.0001). The quality of *θ* oscillations (Cr) recorded in p75^+/+^ and p75^−/−^ mice remains similar between both strains (p75^+/+^: 0.6 ± 0.02, *n* = 24; p75^−/−^: 0.7 ± 0.01, *n* = 31, *p* = 0.1370). Data are presented as mean ± SEM. *P < 0.05, **p < 0.01, and ****p < 0.0001 compared to p75^+/+^ mice.

To study the effect of p75^NTR^ signaling loss on cholinergic receptor-driven oscillatory rhythms in the murine hippocampus, we performed LFP recordings in the CA3 region of horizontal hippocampal slices. LFP gamma oscillations were elicited by applying 20 μM CCh to the bath solution and allowed to stabilize for no <20 min. CCh has been shown previously to be able to induce both gamma (30–80 Hz) and theta (5–10 Hz) oscillations in vitro^34,41–44^. In our experiments, we were able to record large and consistent gamma oscillations (Fig. 1A, top traces). Theta oscillations were apparent after filtering the LFP signal (Fig. 1A, bottom).

We found that gamma power was significantly increased in p75^−/−^ mice compared to p75^+/+^mice (Fig. 1A, B, D). Interestingly, we also observed a significant leftward shift in the peak frequency of the p75^−/−^ power spectra (Fig. 1B, E), suggesting that gamma oscillations in these mice are slower. This finding was further confirmed by performing a gamma oscillation autocorrelation, which showed a shift of the p75^−/−^, compared to gamma p75^+/+^–gamma signal (Fig. 1B). Gamma autocorrelation analysis showed that p75^−/−^ mice display a more rhythmic gamma oscillation compared to p75^+/+^ littermates, which is evident from the greater peak-to-trough distance (Fig. 1B). The increased gamma rhythmicity was confirmed when calculating the coefficient of rhythmicity (Cr), which was significantly increased for gamma oscillations induced in p75^−/−^ hippocampal slices (Fig. 1F). No differences in theta power, peak frequency, or Cr were found between p75^+/+^ and p75^−/−^ mice (Fig. 1A, C–F).

Given that p75^−/−^ mice display slower gamma oscillations, we considered the possibility that this could have an effect on the phase–phase modulation between the gamma and theta rhythms. We observed that gamma peaks from p75^+/+^ were more uniformly distributed around the theta cycle compared to p75^−/−^ (Fig. S4A–D). Interestingly, p75^−/−^ mice appear to have a narrower fit for a Gaussian-like distribution evidenced by a significant increase of the resultant vector of gamma-peak distribution (Fig. S4A–E). Distributions were also different with regard to the gamma-peak-preferred theta phase angle (Fig. S4F). Slower gamma in p75^−/−^ mice was further confirmed when calculating the average number of gamma peaks per theta cycle in both mouse strains (Fig. S4A, C, G).

### Ablation of p75^NTR^ strengthens gamma–theta nesting

**Fig. 2:**
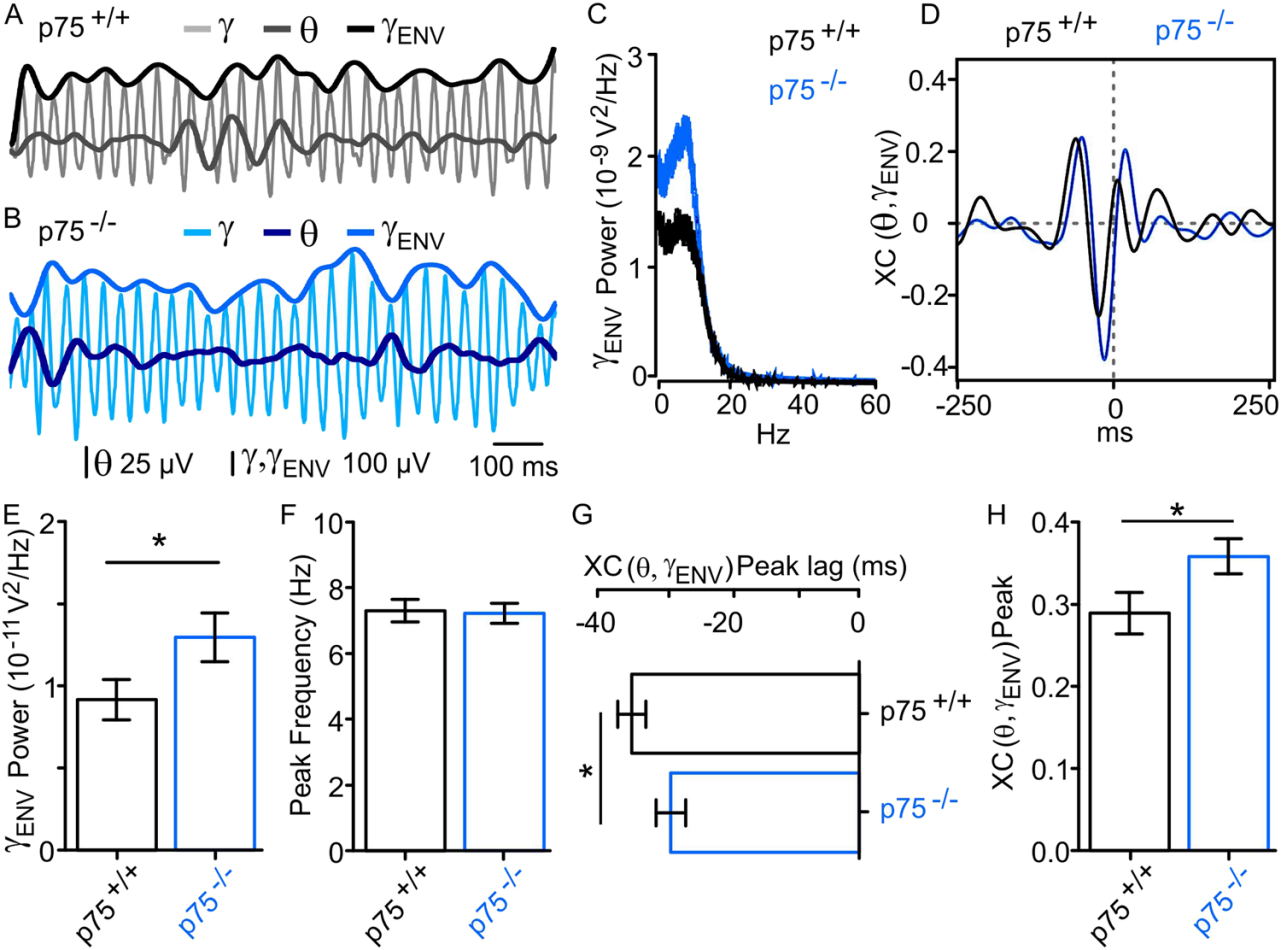
p75^NTR^ ablation strengthens gamma nesting within the theta rhythm. **A**, **B** Representative traces of CCh-induced LFPs in the same experimental conditions as in Fig. 1 showing gamma (*γ*) and underlying theta (*θ*) oscillations. An envelope signal was adjusted to the amplitude of each *γ* peak (*γ*_ENV_, see “Methods”) on recordings from p75^+/+^ (*γ*_ENV_: top black trace; *γ*: gray; *θ*: dark gray) and p75^−/−^ (*γ*_ENV_: top blue trace; *γ*: blue; *θ*: dark blue) mice. **C** Representative power spectra of the *γ*_ENV_ oscillation of p75^+/+^ (black) and p75^−/−^ (blue), showing that this oscillation falls within the frequency range of *θ* rhythm (5–10 Hz) in both mouse strains with similar peak frequency and increased power in p75^−/−^ mice. **D** Cross-correlation function of *θ* with *γ*_ENV_ (XC(*θ*,*γ*_ENV_)) obtained from the same data as in (**A**, **B**) reflects the coincidence of *γ* bouts within the *θ* range with the trough of *θ* as a central prominent negative peak close to zero. The slight lag shift of XC(*θ*,*γ*_ENV_) peak in p75^+/+^ (black) compared to p75^−/−^ (blue) mice reveals the difference of *θ* − *γ*_ENV_ phase locking quantified in (**F**) similar to that observed in *γ* − *θ* phase locking (refer to Fig. S4). **E** Power of *γ*_ENV_ in the *θ* frequency band shows a significant increase in the amplitude modulation of p75^−/−^–gamma oscillations (p75^+/+^: 9.2 ± 1.2 × 10^−11^ V^2^, *n* = 24 vs. p75^−/−^: 13 ± 1.5 × 10^−11^ V^2^, *n* = 31, *p* = 0.0429). **F** Peak frequency of the spectra as in (**C**) from all slices recorded showing that the amplitude modulation of *γ* is oscillating within the *θ* range in both mice strains (p75^+/+^: 7.3 ± 0.3 Hz, *n* = 24 vs. p75^−/−^: 7.2 ± 0.3 Hz, *n* = 31, *p* = 0.3988). **G** Quantification of the XC(*θ*,*γ*_ENV_) peak lag in both mouse strains showing the phase relationship between *θ* and *γ*_ENV_ signals (p75^+/+^: −34.8 ± 6.1 ms, *n* = 24 vs. p75^−/−^: −28.8 ± 4, *n* = 31; *p* = 0.0332). **H** Larger XC(*θ*,*γ*_ENV_) peak in p75^−/−^ mice also indicates a stronger modulation of *γ* amplitude by the underlying *θ* rhythms in p75^−/−^ mice (p75^+/+^: 0.29 ± 0.03, *n* = 24 vs. p75^−/−^: 0.36 ± 0.02, *n* = 31; *p* = 0.0206). Data are presented as mean ± SEM. *P < 0.05 compared to p75^+/+^ mice.

It has been reported previously that gamma rhythm amplitude can wax and wane in the theta range^37^. Although the theta power obtained after filtering the raw signal was not significantly different between p75^+/+^ and p75^−/−^ mice, we tested whether gamma–theta nesting occurs in vitro and whether it was affected by the absence of p75^NTR^ signaling. We proceeded to fit an envelope signal to the peaks of the gamma rhythm, *γ*_ENV_ (see “Methods,” Fig. 2A, B). The spectral analysis of the *γ*_ENV_ showed that in both mouse strains this waxing and waning oscillation resides within the theta-frequency band (Fig. 2C, D). It was interesting to observe that despite the theta peak frequency of *γ*_ENV_ remained the same, there was a significant increase in the γ_ENV_ power in the absence of p75^NTR^ signaling (Fig. 2E, F).

To further assess the strength of the nesting, we performed an XC analysis of both signals (XC(*θ*,*γ*_ENV_)). The XC(*θ*,*γ*_ENV_) displayed a prominent negative peak with a lag close to zero (Fig. 2D), which is indicative of a strong phase locking with the maximum amplitude of gamma oscillations centered at the trough of the theta oscillations^37^ in both mouse strains (Fig. 2A, B, D). However, a difference in the phase relationship between the two signals was evidenced by a significant leftward shift of the XC(*θ*,*γ*_ENV_) in the p75^+/+^ mice indicating that peaks of *γ*_ENV_ occur earlier in the theta cycle in p75^−/−^ littermates (Fig. 2D, G) as has been reported to happen in vivo in rat area CA1 of the hippocampus^37^. The magnitude of the XC(*θ*,*γ*_ENV_) peak was also analyzed in order to determine the strength of the theta–gamma coordination. Compared to p75^+/+^, p75^−/−^ mice showed a significant increase in the XC(*θ*,*γ*_ENV_) peak (Fig. 2D, H), indicating a stronger nesting of gamma within the theta rhythm in the absence of p75^NTR^ signaling.

### p75^−/−^ mice exhibit increased firing synchrony of FSNs during gamma oscillations in the hippocampal network

Cholinergic terminals project to many regions of the hippocampus targeting both CA1 and CA3, where they establish synaptic contacts with a myriad of cell types including GABAergic interneurons. Because of that, tonic cholinergic excitation of interneurons, coupled with their phasic septal GABAergic inhibition, has been suggested to be responsible for the rhythmic discharge of hippocampal interneurons^45^. With the aim to identify the cellular components underlying the observed behavior of neuronal network rhythms and interaction, we performed concomitant recordings of FSN firing and ongoing gamma oscillations induced by 20 μM CCh.

**Fig. 3:**
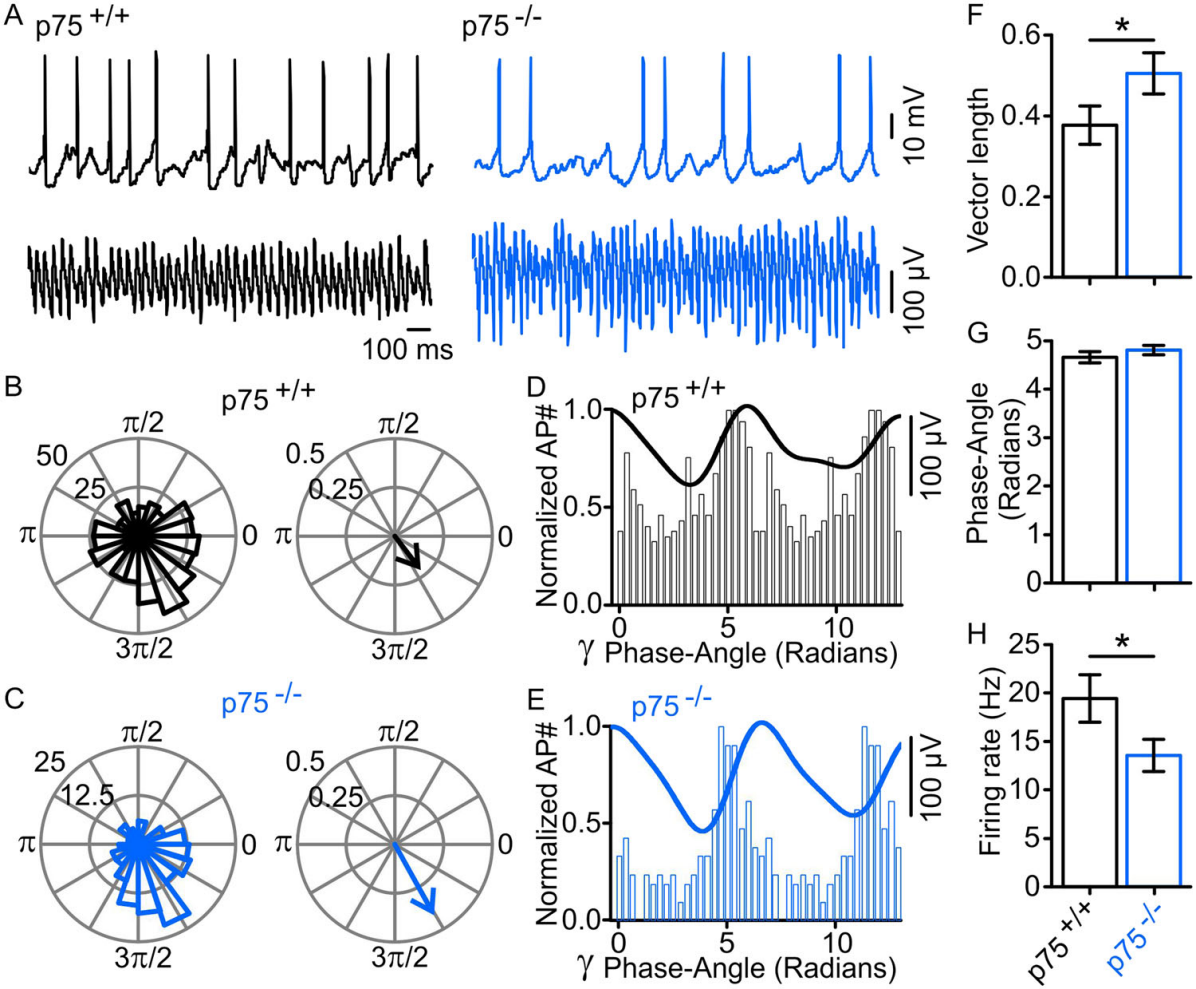
AP firing window of fast-spiking interneurons in p75^+/+^ and p75^−/−^ mice. **A** Representative traces of AP firing from fast-spiking interneurons were recorded concomitantly with gamma oscillations (*γ*) in area CA3 20 min after 20 µM CCh. **B** Polar plots showing the distribution of APs within the *γ* cycle in p75^+/+^ mice. Note that a narrower firing distribution resulting in a larger vector in p75^−/−^ mice (**C**) denotes a higher firing synchronization compared to the firing behavior of FSNs in p75^+/+^ mice. **D** Normalized AP distribution of two *γ* cycles showing firing coincidence with a representative sample trace from p75^+/+^ and p75^−/−^ (**E**) recordings, also showing a tightened firing window in p75^−/−^ strain (better AP synchronization). **F** Resultant vector length quantification (p75^+/+^: 0.38 ± 0.05, *n* = 32; p75^−/−^: 0.51 ± 0.05, *n* = 29, *p* = 0.0240). **G** No significant differences were found in the preferred phase angle between both mouse strains (p75^+/+^: 4.7 ± 0.1 radians, *n* = 32; p75^−/−^: 4.8 ± 0.1 radians, *n* = 29, *p* = 0.3505). **H** Firing rate quantification (p75^+/+^: 19.4 ± 2.5 Hz, *n* = 32; p75^−/−^: 13.5 ± 1.7 Hz, *p* = 0.0281). Data are presented as mean ± SEM. *p < 0.05 compared to p75^+/+^ mice.

Consistent with the higher regularity of gamma oscillations observed in p75^−/−^ mice, we found that p75^−/−^-FSNs are more entrained into gamma oscillations than FSNs in p75^+/+^ littermates (Fig. 3). This was evidenced by a narrower FSN-AP firing distribution around a gamma-preferred phase angle, which resulted in a significantly increased vector length (Fig. 3A–F). It confirms that FSNs fire more synchronized to the gamma rhythm in the absence of p75^NTR^ signaling. However, the preferred phase angle was similar between both mouse strains (Fig. 3G), while the firing rate was lower in p75^−/−^ hippocampal CA3 FSNs (Fig. 3H).

In addition to the increase in FSN firing phase locking to the gamma rhythm, we also assessed excitatory postsynaptic currents (EPSCs) in FSNs during ongoing gamma oscillations in order to study the effect of p75^NTR^ signaling loss on the excitatory drive onto this neuronal class (Fig. S5). We found that EPSCs exhibit larger amplitudes in p75^−/−^ compared to p75^+/+^, while no differences were found for EPSC frequency (Fig. S5A–F). Consequently, EPSC charge transfer was higher in p75^−/−^ compared to p75^+/+^ (Fig. S5F).

Interestingly, we observed that EPSC input to FSNs in both mouse strains was also oscillating in the gamma range with a peak frequency in accordance to the frequency of the extracellular gamma oscillation (Fig. S5G, J). To test if there were differences in the rhythmicity in the oscillatory pattern of the EPSC input, we calculated the Cr and found that p75^−/−^-EPSCs displayed a higher rhythmicity compared to p75^+/+^-EPSCs (Fig. S5H, K, M). To further test the relationship between the oscillatory pattern of the excitatory input to FSNs and LFP gamma oscillations, we performed an XC analysis between the filtered EPSC and LFP signals. We found that while XC lags were similar in both mouse strains, the XC peak showed an increase in p75^−/−^ mice (Fig. S5I, L, M). These data are consistent with our previous results suggesting that the ablation of the p75^NTR^ signal increases the synchronicity of the neural network activity in the mouse hippocampus.

### Ablation of p75^NTR^ signaling prevents Aβ-induced degradation of gamma oscillations in the hippocampal network

It has been observed that AD patients and AD mouse models display alterations of cognition-relevant brain rhythm properties and dynamics^46–48^, prior to plaque formation when only small amounts of soluble forms of Aβ can be detected. We have previously reported that acute Aβ application to hippocampal slices induces a drastic degradation of KA-induced gamma oscillations in area CA3^31^. Furthermore, we have characterized in a novel *App* knock-in AD mouse model^49^ a progressive degradation of gamma oscillations over development, preceding Aβ plaque formation (unpublished data).

**Fig. 4:**
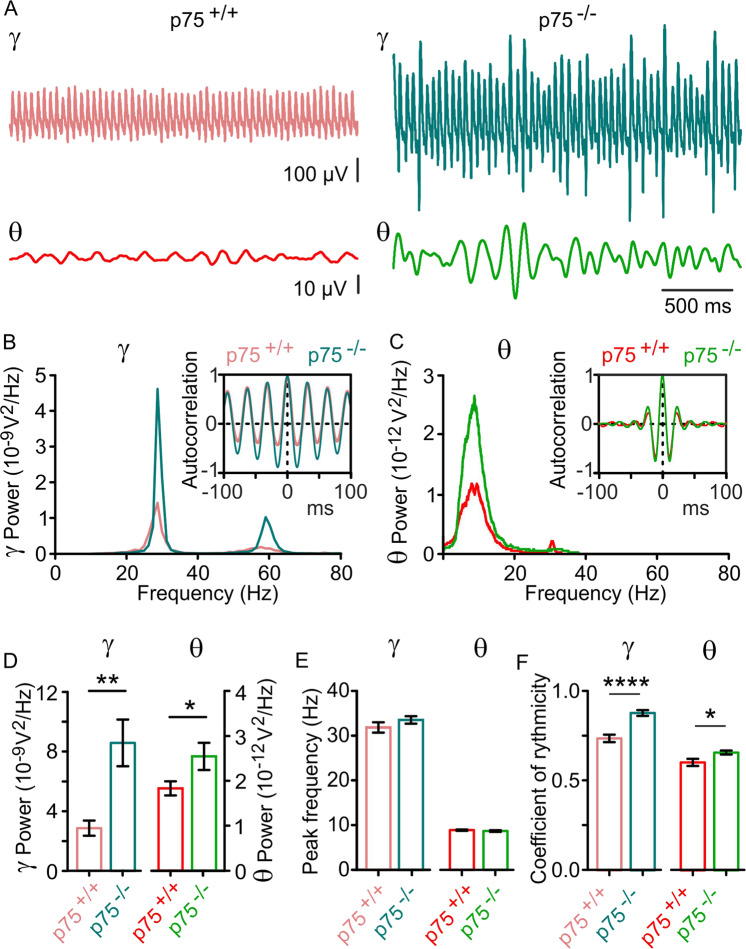
p75^NTR^ ablation prevents Aβ-induced degradation of gamma oscillations. **A** Representative sample traces of CCh-induced gamma oscillations (*γ*, upper traces) and the filtered theta component (*θ*, lower traces) in Aβ-treated hippocampal slices from p75^+/+^ (red) and p75^−/−^ (green) mice 20 min after CCh bath application. **B**, **C** Representative power spectra of *γ* and *θ* oscillations recorded in the experimental conditions described in (**A**). Insets: Corresponding *γ* and *θ* oscillation autocorrelogram. Note that Aβ decreases *γ* and *θ* rhythmicity in p75^+/+^ mice compared to p75^−/−^ where rhythm quality remains unaltered. **D** Mean integrated power of *γ* oscillations (20–80 Hz) and filtered θ (4–10 Hz) from experiments described in (**A**), showing a significant degradation of *γ* and *θ* power induced by Aβ in p75^+/+^ mice (red). **E** Peak frequency of *γ* and *θ* spectra. **F** Coefficient of rhythmicity reveals a significant degradation of *γ* and *θ* rhythms recorded in p75^+/+^ mice, while Aβ failed to degrade rhythms in p75^−/−^. Data are presented as mean ± SEM. *p < 0.05, ***p < 0.005, and ****p < 0.0001 compared to p75^+/+^ mice.

Here, we aimed to test whether the increased stability of cognition-relevant neuronal network dynamics caused by p75^NTR^ ablation could be affected by the presence of Aβ. For this purpose, we recorded CCh-induced gamma oscillations from slices preincubated for 15 min with 50 nM Aβ. Similar to previous findings in WT mice^31,33^, the presence of Aβ led to a significant decrease of gamma oscillation power in p75^+/+^ mice (37.8%, Table S1), while the same treatment failed to reduce gamma power in p75^−/−^ littermates (Table S1 and Fig. 4A, B, D). This preventative effect caused by p75^NTR^ ablation was accompanied by prevention of the reduction of gamma oscillation rhythmicity since Aβ had no effect on Cr in p75^−/−^ mice, while p75^+/+^ littermates showed a significant decrease of gamma rhythm quality (Table S1 and Fig. 4B, F). Peak frequency analysis showed that Aβ caused an increase in the principal frequency in the absence of p75^NTR^ signaling, while the peak frequency in p75^+/+^ mice remained unaltered (Table S1 and Fig. 1B, E vs. Figure 4B, E). Interestingly, theta oscillation power was significantly reduced in p75^+/+^ slices pretreated with Aβ (32.1% of decrease), while p75^−/−^ mice did not show any disruption of the theta rhythm following exposure to Aβ (Table S1 and Fig. 4A, C, D).

### Ablation of p75^NTR^ signaling prevents Aβ-induced disruption of gamma–theta rhythm interaction

Next, we wondered whether exposure to Aβ could affect the observed strong phase locking of the gamma rhythm to a preferred theta phase. By performing the same analysis described in Figure S4, we found that following Aβ preincubation neither p75^−/−^ nor p75^+/+^ showed a significant decrease of the resultant vector length (Table S1 and Fig. S6A–E). However, the preferred phase angle was significantly shifted in p75^+/+^ mice (Table S1 and Fig. S6B, D, F), indicating that exposure to Aβ led to the occurrence of gamma bouts much earlier in the ascending theta phase in p75^+/+^ mice. Consistent with previous findings, Aβ exposure induced an increase in the number of gamma peaks/theta cycle in p75^−/−^ mice, while this relation remained unaltered in p75^+/+^ mice (Table S1 and Fig. S6G). Thus, Aβ induced a shift towards faster gamma oscillations in p75^−/−^ mice without affecting gamma–theta phase locking or gamma oscillation power and rhythmicity.

**Fig. 5:**
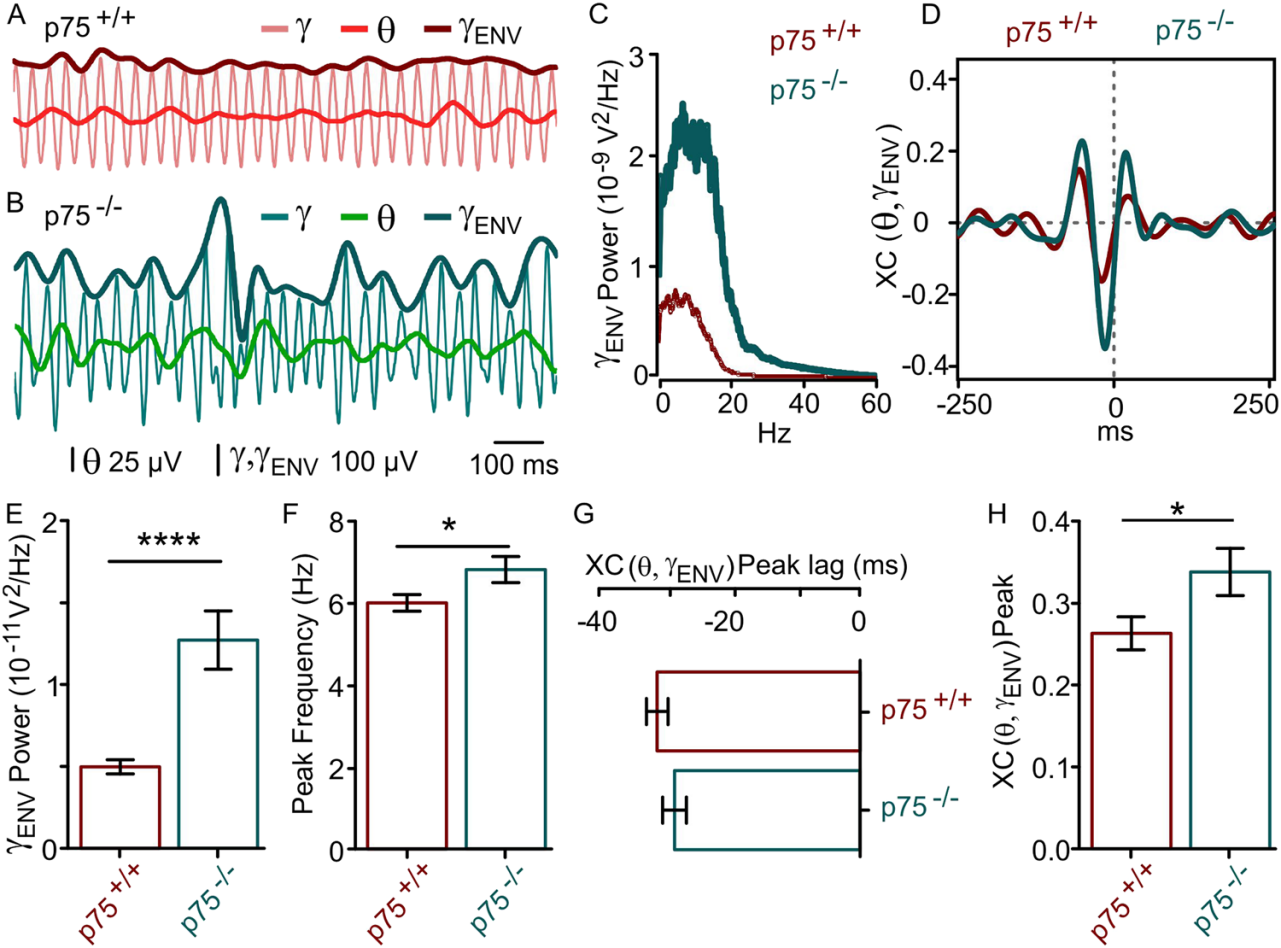
p75^NTR^ ablation prevents Aβ-induced disruption of gamma–theta nesting. **A** Representative sample trace of CCh-induced LFPs after Aβ exposure showing a marked reduction of the envelope signal *γ*_ENV_ (see “Methods”; dark red), *γ* (light red), and *θ* (red) signals in p75^+/+^ mice. **B** Representative sample trace of CCh-induced LFPs after Aβ exposure showing no reduction of the envelope signal *γ*_ENV_ (dark green), *γ* (light green), and *θ* (green) signals recorded in p75^−/−^ mice. **C** Representative power spectra of the *γ*_ENV_ signal from p75^+/+^ (dark red) and p75^−/−^ (dark green), showing that Aβ dramatically decreases *γ*_ENV_ power in p75^+/+^ mice. **D** Cross-correlation function of *θ* with *γ*_ENV_ (XC(*θ*,*γ*_ENV_)) obtained from the same data as in (**A**, **B**) showing that Aβ significantly affects the magnitude of the *θ* and *γ*_ENV_ cross-correlation. **E** Mean *γ*_ENV_ power within the *θ* frequency band shows that Aβ drastically decreases *γ* amplitude modulation of p75^+/+^ *γ* oscillations, while *γ*_ENV_ power of p75^−/−^ mice remains unaltered. **F** Peak frequency of the spectra as in (**C**) from all slices recorded showing that the *γ* amplitude modulation is oscillating within the *θ* range in both p75^+/+^ and p75^−/−^ mice. **G** Quantification of the XC(*θ*,*γ*_ENV_) peak lag in both mouse strains showing the phase relationship between *θ* and *γ*_ENV_ signals. **H** The larger XC(*θ*,*γ*_ENV_) peak in p75^−/−^ mice signifies a stronger modulation of *γ* amplitude by the underlying *θ* rhythm, indicating that p75^NTR^ ablation prevents the Aβ-induced impairment of *θ*–*γ* nesting observed in p75^+/+^ mice. Data are presented as mean ± SEM. *P < 0.05 and ****p < 0.0001 compared to p75^+/+^ mice.

Thereafter, we investigated to what extent Aβ might alter the nature of the nesting of both rhythms. For this purpose, we recorded CCh-induced gamma oscillations from hippocampal slices from both p75^+/+^ and p75^−/−^ mice 15 min after preincubation with Aβ and performed XC and spectral analysis of the envelope signal fitted to gamma peaks. As previously observed, the spectral analysis of *γ*_ENV_ fitted to gamma peaks showed that in both mouse strains this oscillation resides within the theta-frequency band (Fig. 5A–C). Interestingly, in p75^+/+^ mice Aβ caused a slowing of *γ*_ENV_ compared to p75^−/−^, where *γ*_ENV_ peak frequency remained unaltered (Table S1 and Fig. 5C, F). Notably, in p75^+/+^ mice Aβ caused a 45.7% decrease of *γ*_ENV_ power, while *γ*_ENV_ power was not affected by Aβ in the absence of p75^NTR^ signaling (Table S1 and Fig. 5C, E).

To further elucidate the phase relationship between both *θ* and *γ*_ENV_, we assigned the magnitude and lag of the central peak of the XC (XC(*θ*,*γ*_ENV_)) as measurements of the interaction strength and the phase locking of both signals, respectively^37^. Although Aβ did not induce a significant shift in the phase relationship between *θ* and *γ*_ENV_ signals (Table S1 and Fig. 5D, G), the XC(*θ*,*γ*_ENV_) peak of the signals recorded from p75^+/+^ was significantly decreased by Aβ exposure, while the XC(*θ*,*γ*_ENV_) peak of the signals recorded from p75^−/−^ remained unchanged after Aβ exposure (Table S1 and Fig. 5D, H).

## Discussion

### New insights into the role of p75^NTR^ signaling in hippocampus physiology

Although some effects on the survival and proliferation of BFCNs after the ablation of neurotrophin receptors have been described before^50–53^, the consequences of these effects for the cognition-relevant network dynamics of one of the major targets of BFCN output—the hippocampal formation—are not known. Noteworthy, TrkB receptor deficiency in FSNs has been shown to decrease the power of gamma oscillations in the hippocampal network^21^, suggesting that disturbing neurotrophic signaling can have an impact on neuronal network dynamics in the hippocampus.

p75^NTR^ knockout mice exhibit an increase in cholinergic cell number and size in the basal forebrain, but also an increase in the number of cholinergic axonal fibers targeting the hippocampus, as well as an increased choline acetyltransferase activity^50,54^. In the present study, we report for the first time that this increase of cholinergic input onto the hippocampus has a cognition-relevant physiological consequence. We describe a marked increase in the power of CCh-induced gamma oscillations in area CA3 hippocampal of p75^−/−^ mice, which is consistent with histochemical studies. In addition to the increased gamma oscillation power, gamma activity was also more rhythmic in p75^−/−^ mice, most likely caused by the observed increased synchrony of FSN activity. A further physiological consequence is the stronger interaction between gamma and theta oscillators, which was evidenced by an increased phase lock of the gamma rhythm to the underlying theta rhythm. Our functional findings support the idea that p75^NTR^ plays a key role in the pruning of hippocampal cholinergic innervation, at the very least in the early stages of development^25,55^, and has a strong impact on cognition-relevant neuronal network dynamic in the hippocampus. Our data provide the basis for further studies into the nature of these rhythms interaction in vitro as well as in vivo in the absence of p75^NTR^ signaling.

### Ablation of p75^NTR^ signaling increases gamma–theta rhythm coupling

Theta oscillations have been reported to interact with and change the properties of the gamma rhythm, such as amplitude, phase, or power of the oscillation^4–6,8,11,12,56^. Although other studies have reported large theta oscillations in vitro^57–59^, in our brain slice preparations theta oscillations were more modest. This is very likely due to fundamental differences in the experimental procedures such as the cutting orientation of the slices (horizontal rather than transverse to generate robust gamma oscillations) or the fact that some studies use cocktails of receptor agonists and antagonists to elicit theta oscillations^57^, while others use whole hippocampal preparations to study spontaneously generated theta rhythms^58^.

Nonetheless, even though our experiments were designed to favor the recording of gamma oscillations, we were able to obtain a small but consistent underlying theta rhythm that could be isolated by filtering the LFP signal in order to remove the dominant gamma oscillations (see theta Cr values in Figs. 1 and 5). This allowed us to study the distribution of gamma peaks to the instantaneous theta phase as well as the nesting of gamma in the underlying theta rhythms. To our knowledge, our study is the first to show that CCh application (20 µM) can induce gamma and theta rhythms that coexist and are phase-locked in area CA3 of an acute hippocampal slice preparation. Therefore, our results add relevant evidence to the notion that an intrinsic theta oscillator exists in the hippocampus^16,60^.

In our study, the spectral analysis of LFP gamma oscillations in p75^−/−^ mouse networks showed a leftward shift of the peak frequency, which suggested that there is a slowing of the gamma rhythm in the absence of p75^NTR^ signaling. Indeed, gamma autocorrelograms and gamma peaks/theta cycle analysis (see Fig. S4) corroborated this slowing phenomenon. Notably, the preferred theta phase angle was different for each of the two mouse strains. Altogether, these factors may effectively alter the phase–phase interaction of theta and gamma oscillations. In fact, the distribution analysis of gamma peaks within a theta cycle showed a stronger preference (locking) of gamma to the preferred phase angle of the underlying theta wave when p75^NTR^ signaling is absent (see Fig. S4). Given that no significant spectral differences of the theta rhythm were found between both mouse strains, it seems unlikely that the theta oscillation was the main driver of the increased phase locking. Instead, our data suggest that this stronger gamma-phase coupling arises from alterations of the cellular mechanisms that control the dynamics of hippocampal rhythms.

It has been proposed that such an underlying cellular mechanism could be the enhancement of the precision of AP firing timing within a slow oscillation (i.e., theta) cycle by fast precisely tuned hyperpolarizations of the membrane potential^61^. This principle appears to be particularly relevant in hippocampal local circuits where interneurons, especially FSNs, play a central role in determining the firing rate and phase of pyramidal cells during ongoing gamma oscillations^62^. This proper timing is critical for maintaining network stability. In fact, activation of FSNs powerfully controls the PC network and rhythm generation optimally at 8 Hz, while silencing them disrupts an intrinsic theta rhythm in CA1^63^. Interestingly, on the cellular level, we found that FSNs from p75^−/−^ mice displayed a more synchronized activity during the gamma rhythm compared to p75^+/+^ FSN firing activity, and with no difference in the gamma-preferred p hase angle. This better synchronized activity of FSNs likely accounts for the differences observed between both mouse strains since a neural network more entrained to the gamma rhythm will be also more attuned to the underlying theta rhythm as is the case in p75^−/−^ hippocampus with better synchronized FSN activity. On the other hand, a larger and more gamma-locked excitatory input to FSNs has been observed in p75^−/−^ mice, while an overall decrease of excitability was apparent. The most plausible interpretation of these findings may be found in an enhancement of the postsynaptic computational capacity of FSNs to respond to glutamatergic inputs from pyramidal neurons in a situation where the mechanism of excitatory event integration is more synchronized (see Fig. S5).

In addition, we also tested whether the theta-attuned gamma oscillations (here induced by CCh) also exhibit an oscillating amplitude peak patterned in the theta band as it occurs in vivo^37^. Our data showed gamma–theta nesting in both mouse strains with better coupling of the rhythms in the absence of p75^−/−^ signaling. Moreover, p75^−/−^ mice displayed a larger-amplitude oscillation. This opens the possibility for the hippocampal microcircuitry to resonate without a larger external theta input, which could lead to an increase in gamma oscillation power via a mechanism similar to the one operating in the intact brain. Studies directed to dissect the modulation phenomena will further our understanding of the theta–gamma interactions observed here. It is known that the inputs from the GABAergic inhibitory neurons from the MS/DBB—regarded as the main theta input into the hippocampus—can greatly influence this modulation^16,17,60,64^. In line with the better synchronization with the gamma rhythm observed for the FSN firing behavior in p75^−/−^ mice, it has been proposed that the amplitude modulation of the gamma rhythm at theta frequencies may arise from a phasic interruption of the interneuronal network activity, as the gamma power and interneuronal firing are both modulated at theta and are in phase with each other^1^.

Taken together, and consistent with previous reports^17,65,66^, both gamma–theta phase locking/nesting and gamma oscillation strengthening in the presence of CCh strongly support the notion that the role of the cholinergic innervation of the hippocampal region—increased due to p75^NTR^ ablation—is to facilitate the coordination between the gamma rhythms generated in the hippocampus and GABAergic-driven theta rhythms generated also in the MS/DBB.

### Functional implications of p75^NTR^ signaling modulation for AD

Normal neuronal synchrony underlies the generation of brain rhythms, such as gamma oscillations, that promote cognitive functions. There is increasing evidence that the disruption of these rhythms can be used as an early functional biomarker of AD since initial alterations in hippocampal network activity arise long before Aβ accumulation and plaque formation. Both disruption of brain rhythms and aberrant Aβ accumulation constitute the pathophysiological and histological hallmarks of the disease, respectively. Moreover, these disease features correlate with the progressive cognitive decline patients experience with the advance of AD to ever more severe stages. Supporting evidence comes from animal studies in which robust alterations of theta–gamma coupling in area CA1/subiculum of AD mouse models was observed before Aβ overproduction^47,67^.

We have also observed that acute exposure of neuronal networks to Aβ induces firing desynchronization and degradation of hippocampal gamma oscillations in vitro^31,33,36^. Consistent with previous findings, here we have observed that acute Aβ induced degradation of gamma oscillations and rhythmicity in p75^+/+^ mice. Theta oscillation power was also decreased, and a significant disruption of theta–gamma nesting was evident in area CA3 of p75^+/+^ hippocampal slices. It is noteworthy that the rhythmicity of theta oscillations also significantly decreased compared to p75^−/−^ Aβ-treated slices. Given the drastic disruption of both rhythms and their interaction, it is tempting to speculate that the Aβ-induced shift of the gamma-preferred theta phase angle (see Fig. S6 and Table S1) may be responsible for the observed gamma rhythm slowing and decrease of the faster and larger gamma bouts within the theta range when p75^NTR^ signaling is intact.

p75^NTR^ promotes Aβ neuritic dystrophy both in vivo and in vitro^28^, and is upregulated in the hippocampus of late-stage AD patients^68^. This upregulation of p75^NTR^ expression in AD patients’ brain parallels the one observed in AD mouse models^69^. Synaptic loss is one of the features of neuronal damage in AD, and it strongly correlates with cognitive impairment in patients^70,71^. Interestingly, the presence of interfering molecules that potentially prevent Aβ–p75^NTR^ interaction has been shown to have neuroprotective properties^69,72^. Moreover, age-dependent degeneration of BCNF is prevented by modulation of p75^NTR^ ^19^.

Highly complementary to these previous studies at cellular and molecular levels, our functional findings reveal that p75^NTR^ ablation significantly strengthens and protects neuronal network dynamics from Aβ-induced neuronal network dysfunction. The p75^−/−^ mouse strain used in our study lacks the extracellular receptor domain capable of interacting with extracellular ligands. As such, it displayed a gamma rhythm with increased power and increased rhythmicity, as well as better synchronized FSN activity. The latter appears to be the profound functional effect caused by the deletion of p75^NTR^ signaling since a more synchronized FSN activity leads to the rescue of gamma oscillations during sharp waves ripples, decreases Aβ load, and induces a turnover of microglia from a pro-inflammatory phenotype towards a phagocytic state in the 5XFAD AD mouse model in vivo^48^. In addition, optogenetically driving medial septal FSNs restores hippocampal slow gamma oscillation amplitude as well as phase-amplitude coupling of the J20 AD mouse model^73^.

Finally, our study identifies p75^NTR^ as a suitable target in the search for neuroprotective and/or neurorestorative strategies against AD that opens treatment avenues for other cognitive-compromised and amyloid-driven pathologies. Studies that include the blockade of p75^NTR^ signaling as a therapeutic step during AD progression might help to counteract the degeneration of cholinergic neurons, altered neuronal network dynamics, and, hence, the cognitive dysfunction typical of AD.

## Supplementary information

Supplementary Information

Supplementary Figure 1

Supplementary Figure 2

Supplementary Figure 3

Supplementary Figure 4

Supplementary Figure 5

Supplementary Figure 6

Supplementary Table 1
