# Supplementary material for: Ablation of p75NTR signaling strengthens gamma–theta rhythm interaction and counteracts Aβ-induced degradation of neuronal dynamics in mouse hippocampus in vitro
Source: Transl Psychiatry. 2021 Apr 9;11:212. doi: 10.1038/s41398-021-01332-8 (PMC8035168; doi:10.1038/s41398-021-01332-8)
Supplement: Supplementary file 1 — Supplementary Information [file 41398_2021_1332_MOESM1_ESM.docx]

**Supplementary figures legend**

**Supplementary Figure 1. Representative PCR analysis of p75^NTR^ alleles.** Three samples showing the presence of a 437 bp amplicon in wild-type (p75^+/+^) mice, a 252 bp amplicon in the knock-out (p75^-/-^) mice and both amplicons in the heterozygous (p75^+/-^) mice.

**Supplementary Figure 2. Electrophysiological characterization of FSNs.**

Example traces of recordings performed in current-clamp mode showing the characteristic firing properties of FSNs contrasted with the firing properties displayed by non-FSNs. Each panel (A-C) shows the firing response (top) to different protocols delivered via the patch pipette (bottom). Typical FSN is shown to the left and non-FSN is shown to the right. **A)** 300 pA depolarizing step triggers irregularly spaced fast spiking action potentials with V-shaped after-hyper-polarizations (AHPs) in FSN while non-FSN display a more regular firing with slow U-shaped. FSN also showed frequent and large excitatory post synaptic potentials in response to -150 pA hyperpolarizing step which were characteristic for this interneuron-class without eliciting an ‘‘I_h_-sag’’. However, non-FSN showed a characteristic large ‘‘I_h_-sag’’ response to the negative step. **B)** FSNs fired throughout the ramp with marked trend to frequency increase while maintaining spike integrity in terms of amplitude and width. Non-FSN failed to properly fire throughout the current ramp with progressive deterioration of the action potential amplitude and width. **C)** 150 pA depolarizing step induces a subtle increase of firing frequency while a subsequent 300 pA additional increase causes a prominent firing frequency increase in FSN. Compared to baseline, initial 150 pA depolarizing step induces a prominent increase of firing frequency in non-FSN while additional depolarizing step (300 pA) did not result in a significant further firing frequency increase as typically observed in FSNs.

**Supplementary Figure 3. Validation of the peak adjustment settings.** Spectral parameters and γ_ENV_ (XC(θ,γ_ENV_)) using peak separation set to 50 and 200 from WT (p75^+/+^) mice. Data is presented as mean ± SEM. Statistics performed Student's t-test (***p<0.005). The analysis of the peak lag was assessed with Mann Whitney test since neither choosing peak criteria adjustment of 50 nor 200 yielded normally-distributed values as in Fig. 2 and Fig. 5 with 100. Overall, it indicates that when the method fails to detect the existing gamma amplitude oscillation within central theta frequencies further analysis reveals spurious interactions in terms of phase and strength. It confirmed that the peak mode set at 100 is the most accurate to reliably detect the existing gamma amplitude oscillation within theta band hence guarantying the detection of the existing *nesting* as it has been described *in vivo*. We consider that it further support moving to deepest analysis of such interaction in CA3 area of the hippocampus *in vitro/ex-vivo* which is highly difficult to assess *in vivo*.

**Supplementary Figure 4. *p75^-/-^ mice exhibit increased phase-lock of gamma oscillations to the underlying theta rhythm*. A)** Representative gamma (γ, black) and theta (θ, dark gray) components from the raw data recorded in p75^+/+^mice. **B)** Polar plots showing the distribution of p75^+/+^ γ peaks within a θ cycle (left) and the resultant vector (right). γ and θ oscillation phases are presented in radians; the peak of the oscillation cycle corresponds to 0 radian and the trough corresponds to π radians (see methods). **C)** Representative γ (blue) and θ (dark blue) components from the raw data recorded in p75^-/-^mice. **D)** As shown in B, polar plots show the distribution of γ peaks within a θ cycle in p75^-/-^ mice (left) and the corresponding resultant vector (right). Note that a faster γ appears earlier in the ascending θ phase in p75^-/-^ compared with p75^+/+^ mice. **E)** Vector length calculated from the unitary vector shown in B and C, evidencing a marked increase of the γ phase-locking to the preferred θ phase in p75^-/-^ mice: p75^+/+^: 6.7 ± 0.81 x 10^-02^ n=24 vs. p75^-/-^: 8.7 ± 0.67 x 10^-02^, n=31, p=0.0360. **F)** Averaged unitary vector phase-angle of each slice resulting from the summation of all phase-angles from p75^+/+^ (black) and p75^-/-^ (blue) showing that γ peaks preferred θ phase is significantly shifted in p75^+/+^compared to p75^-/-^ mice (p75^+/+^: 6.14 ± 0.02 rad, n=24 vs. p75^-/-^: 6 ± 0.04 rad, n=31, p=0.0031). **G)** Mean gamma peak per θ cycle. p75^-/-^ mice have a significant reduction in the number of γ peaks per θ cycle (p75^+/+^: 4.3 ± 0.1 γ peaks/θ cycle, n=24 vs. p75^-/-^: 4 ± 0.1 γ peaks/θ cycle, n=31, p-value= 0.0153), consistent with the slowing of γ observed in Fig. 1. Data is presented as mean ± SEM. * indicates p<0.05, **p<0.01 compared to p75^+/+^ mice.

**Supplementary Figure 5. Excitatory synaptic input to fast-spiking interneurons is increased and more synchronized into gamma rhythm in p75^-/-^ mice. A)** Representative traces of EPSCs (Vh=-70mV) recorded from FSNs and concomitant LFP recordings in p75^+/+^ and p75^-/-^ mice. **B-E)** Cumulative probability (left) and frequency distribution graph (right) of the EPSC amplitude and inter-event interval, respectively, in p75^+/+^ (black) and p75^-/-^ (blue) FSNs. **F)** Summary bar-graphs of p75^+/+^ (black) and p75^-/-^ (blue) EPSC amplitude (p75^+/+^: 28.03 ± 3.17 pA, n=9; p75^-/-^: 52.55±6.37 pA, n=16; p=0.0056; Fig. 5A-C, F), frequency (p75^+/+^: 39.98±3.17 Hz, n=9; p75^-/-^: 36.32±2.07 Hz, n=16; p=0.1623) and charge transfer (p75^+/+^: 214890±37277 pA*ms, n=9; p75^-/-^: 411072±63505 pA*ms, n=16, p=0.0197). **G, J)** Representative power spectra from the LFP signal (p75^+/+^ and p75^-/-^ mice; gray) and EPSCs in p75^+/+^ (black) and p75^-/-^ mice (blue). **H, K)** Representative auto-correlograms from the LFP signal (p75^+/+^ and p75^-/-^ mice; gray) and EPSCs in p75^+/+^ (black) and p75^-/-^ mice (blue). **I, L)** Representative cross-correlation (XC) graphs between the LFP and EPSC signals in p75^+/+^ (black) and p75^-/-^ mice (blue). **M)** Summary bar-graphs of the coefficient of rhythmicity (Cr): p75^+/+^ Cr: 0.3420±0.02875, n=9; p75^-/-^ Cr: 0.4748±0.04195, n=16; p=0.0190; XC lag: p75^+/+^: -6.067±1.139 ms, n=9; p75^-/-^: -5.219±0.3914 ms, n=16; p=0.2005 and XC peak in p75^+/+^ (black) and p75^-/-^ mice (blue): p75^+/+^: -0.4780±0.03680, n=9; p75^-/-^: -0.5924±0.04428, n=16; p=0.0469. Quantifications were performed on 1 min-segments. Unpaired t-test (one-tailed) was used for statistical significance on absolute values. Data is presented as mean ± SEM. * indicates p<0.05 and ** indicates p<0.01 compared to p75^+/+^ mice.

**Supplementary Figure 6. p75^NTR^ ablation prevents Aβ-induced alterations of gamma-theta phase-locking. A)** Representative gamma (γ, red) and theta (θ, light red) components from the raw data recorded in p75^+/+^ mice after Aβ treatment. **B)** Polar plots showing the distribution of gamma peaks within a theta cycle in p75^+/+^ mice (left) and the resulting unitary vector (right). γ and θ oscillation phases are presented in radians; the peak of the oscillation cycle corresponds to 0 radian and the trough corresponds to π radians (see methods). **C)** Representative γ (green) and θ (light green) components from the raw data recorded in p75^-/-^ mice after Aβ treatment. **D)** Polar plots showing the distribution of gamma peaks within a theta cycle in p75^-/-^ mice (left) and the resulting unitary vector (right). **E)** Resulting vector length was not significantly altered by Aβ in either p75^-/-^or p75^+/+^ mice. **F)** Averaged unitary vector phase-angle resulting from the summation of all phase-angles recorded in p75^+/+^ (red) and p75^-/-^ (green) mice showing that Aβ significantly shifts the γ-preferred θ-phase in p75^+/+^compared to control conditions (Table S1). **G)** Mean γ peak per θ cycle. Data is presented as mean ± SEM. ** indicate p<0.01compared to p75^+/+^ mice.

**Supplementary methods**

*Phase-phase relationship (Fig. S3 and S5)*

To detect if any phase-phase relationship the instantaneous phase of gamma and theta oscillations was calculated using a Hilbert transform on the pre-processed LFP recording. Analysis was performed with a Matlab custom-written routine where the peak phase of the gamma oscillation (0 rad) was indexed and binned around its corresponding theta cycle. The unitary vector was calculated by summing the individual unit vector for each angle (a), calculated as u = e^ia^. Since we aimed to determine whether any difference in the distributions could be found, no Rayleigh test was performed to test for uniformity of gamma peak distribution.

For statistic comparisons the absolute value of the resultant vector length was considered as an indicator of phase preference strength. Average number of gamma peaks per theta cycle was calculated as the total number of gamma peaks divided by total number of theta cycles per segment analyzed.

*Excitatory postsynaptic currents analysis (Fig. S4)*

EPSC were recorded in voltage-clamp with membrane potential held at -70 mV and detected off-line using MiniAnalysis software (Synaptosoft, Decatur, GA, USA). Charge transfer, event amplitude and inter-event-interval (IEI) were analyzed using Excel software (Microsoft Office) and GraphPad Prism (GraphPad Software, USA) with the results representing average values taken over 1min periods. Power spectra of the EPSC were calculated using the same 1 min period using Clampfit 10.7.0 software (Molecular Devices) as well as the EPSC Cr and cross-correlation analysis between the LFP and EPSC recordings. In the latter signals were pre-processed using a RC (single pole) bandpass filter set to 15-45 Hz.
