# Supplementary figures and images for: Ablation of p75NTR signaling strengthens gamma–theta rhythm interaction and counteracts Aβ-induced degradation of neuronal dynamics in mouse hippocampus in vitro
Source: Transl Psychiatry. 2021 Apr 9;11:212. doi: 10.1038/s41398-021-01332-8 (PMC8035168; doi:10.1038/s41398-021-01332-8)

600 bp  
500 bp  
400 bp  
300 bp  
200 bp  
100 bp

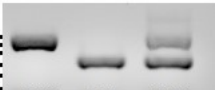

P75<sup>+/+</sup>

P75<sup>+/-</sup>

P75<sup>+/-</sup>

Supplement: Supplementary file 2 — Supplementary Figure 1 [file 41398_2021_1332_MOESM2_ESM.pdf]

FS-IN

Non FS-IN

A

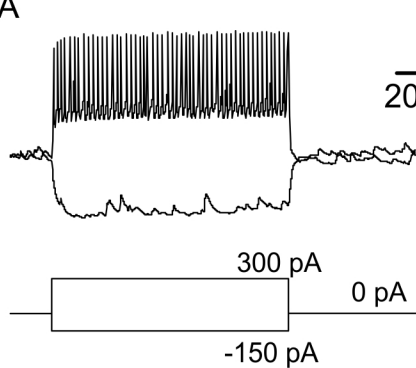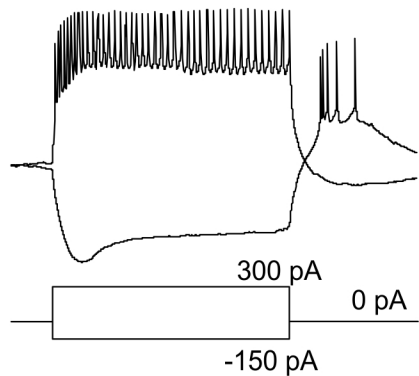

B

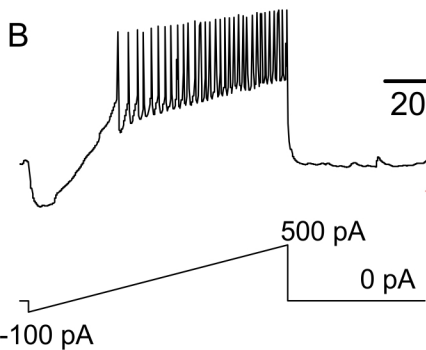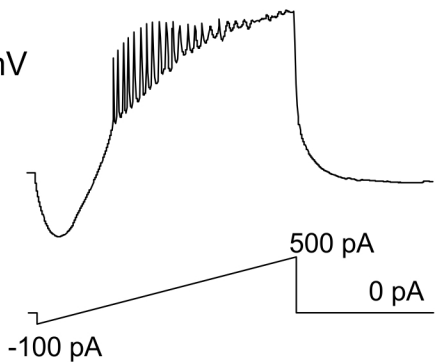

C

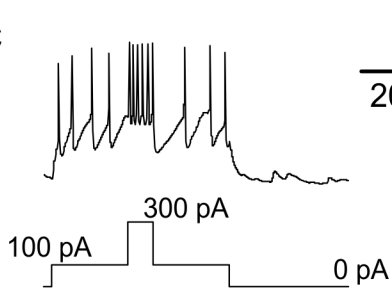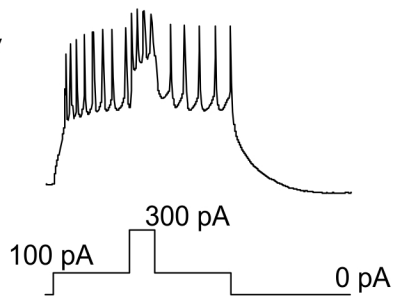

Supplement: Supplementary file 3 — Supplementary Figure 2 [file 41398_2021_1332_MOESM3_ESM.pdf]

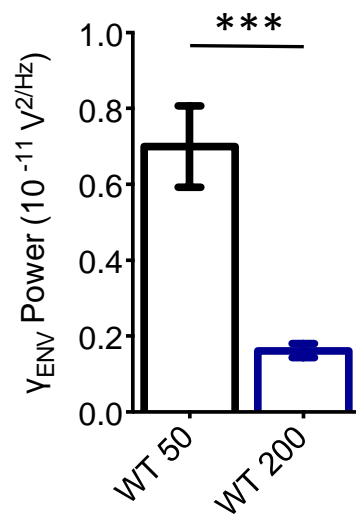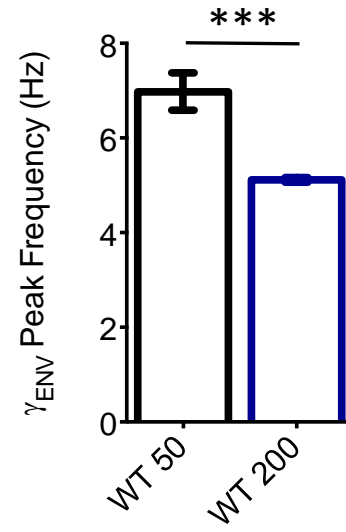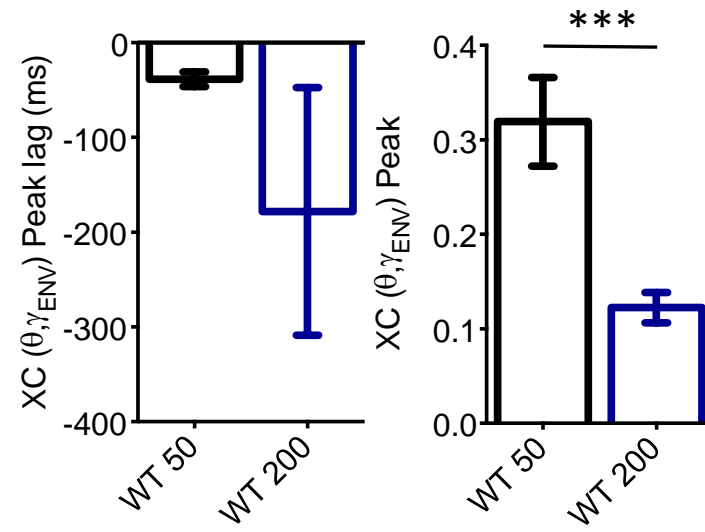

Supplement: Supplementary file 4 — Supplementary Figure 3 [file 41398_2021_1332_MOESM4_ESM.pdf]

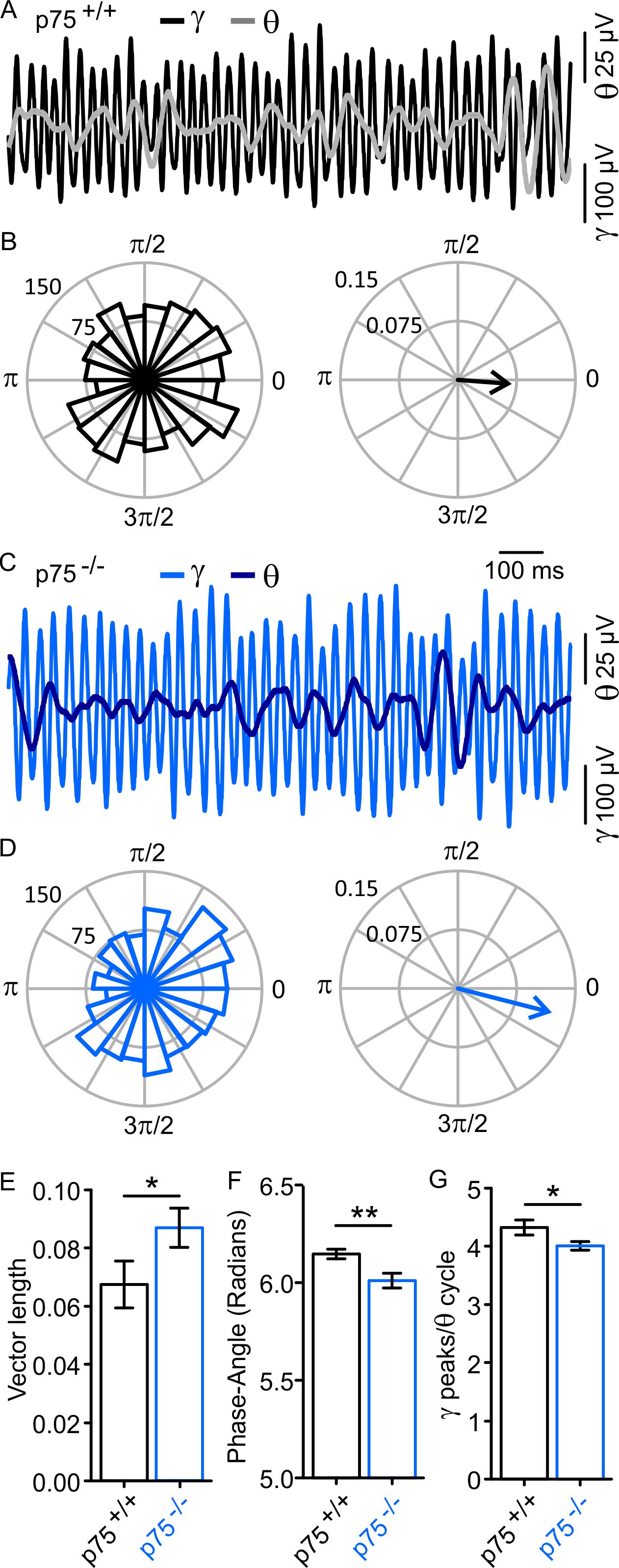

Supplement: Supplementary file 5 — Supplementary Figure 4 [file 41398_2021_1332_MOESM5_ESM.jpg]

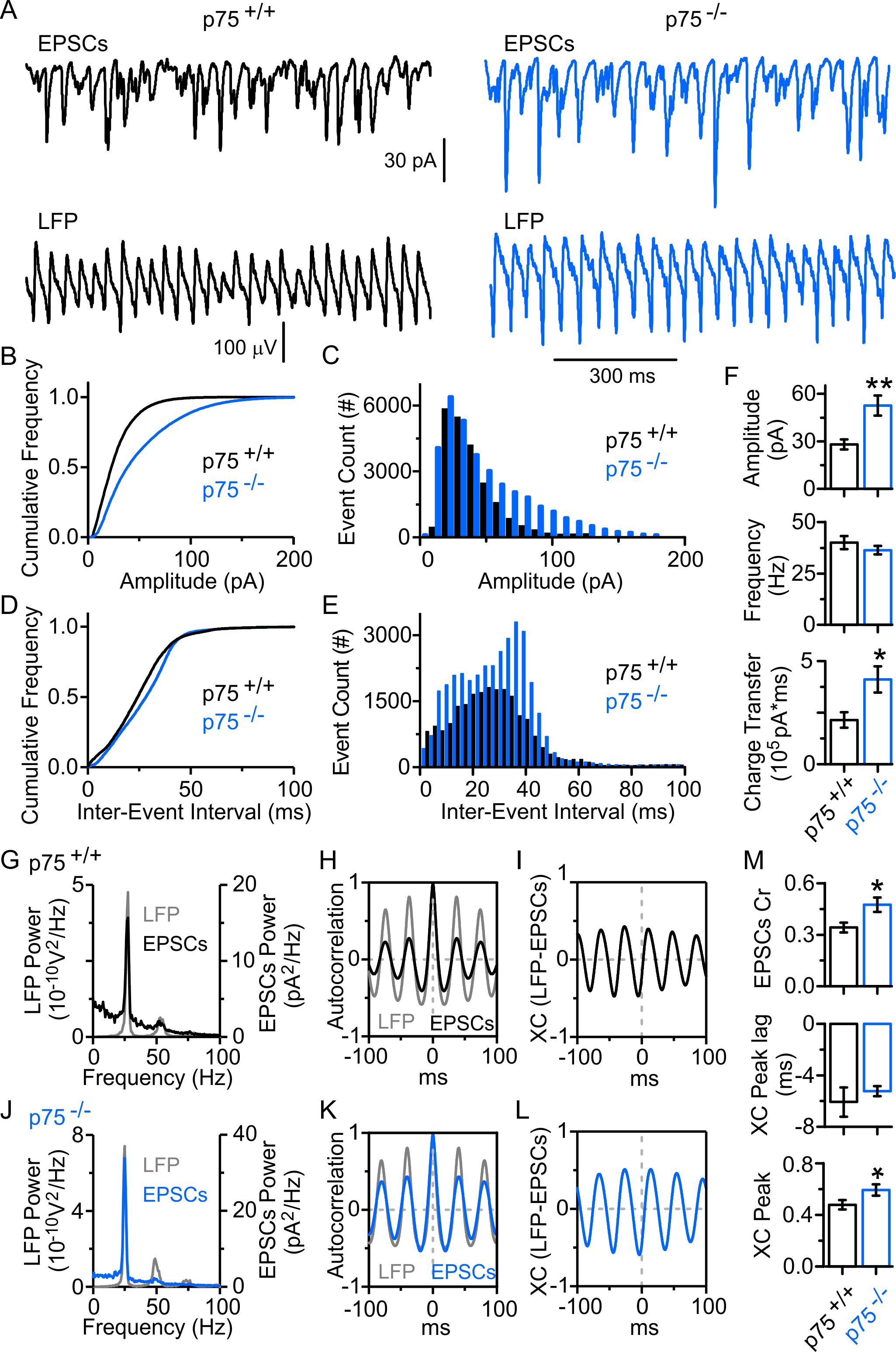

Supplement: Supplementary file 6 — Supplementary Figure 5 [file 41398_2021_1332_MOESM6_ESM.jpg]

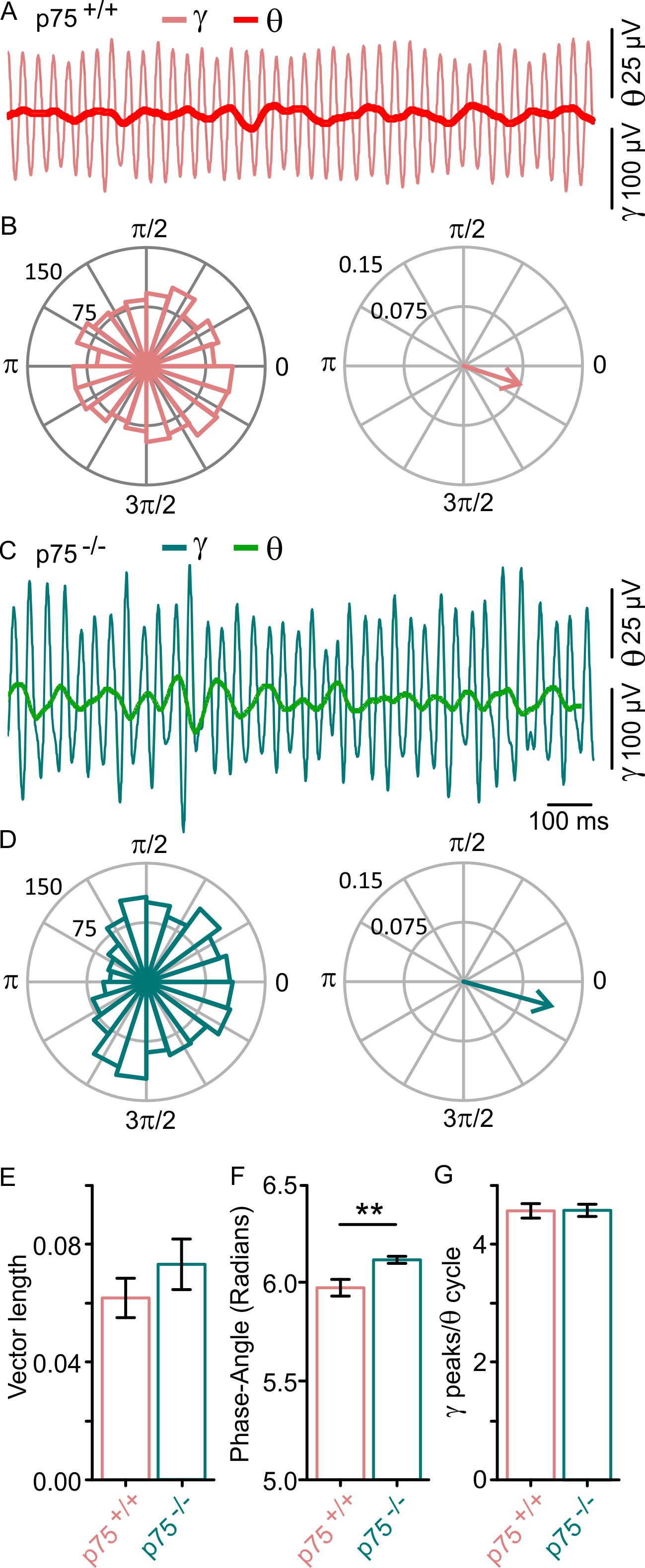

Supplement: Supplementary file 7 — Supplementary Figure 6 [file 41398_2021_1332_MOESM7_ESM.jpg]
