# Supplementary material for: Ablation of p75NTR signaling strengthens gamma–theta rhythm interaction and counteracts Aβ-induced degradation of neuronal dynamics in mouse hippocampus in vitro
Source: Transl Psychiatry. 2021 Apr 9;11:212. doi: 10.1038/s41398-021-01332-8 (PMC8035168; doi:10.1038/s41398-021-01332-8)
Supplement: Supplementary file 8 — Supplementary Table 1 [file 41398_2021_1332_MOESM8_ESM.pdf]

Supplementary Table 1. Summary results of Aβ effects in both mouse strains with their corresponding control groups. S: Student's t test; M: Mann-Whitney test. Data is presented as mean ± SEM. * indicates $p<0.05$, **$p<0.01$, ***$p<0.005$, ****$p<0.0001$.

Parameters		$p75^{+/+}$			
		Control (n=24)	*Aβ (n=25)*		*p-value*
γ power (V^2)		$4.5 \pm 0.78 \times 10^{-09}$	$2.8 \pm 0.5 \times 10^{-09}$	S	0.0381*
γ peak frequency (Hz)		30.9 ± 0.9	32.1 ± 1.2	S	0.2086
γ Coefficient of Rhythmicity		0.8 ± 0.02	0.7 ± 0.02	S	0.0229*
θ power (V^2)		$2.7 \pm 0.37 \times 10^{-12}$	$1.8 \pm 0.15 \times 10^{-12}$	S	0.0105*
θ peak frequency (Hz)		8.9 ± 0.1	8.9 ± 0.2	S	0.4716
θ Coefficient of Rhythmicity		0.6 ± 0.02	0.6 ± 0.02	S	0.1660
γ-θ phase locking	Vector length	$6.7 \pm 0.81 \times 10^{-02}$	$6.2 \pm 0.66 \times 10^{-02}$	S	0.2907
	Phase angle	6.14 ± 0.02	5.98 ± 0.04	M	0.0008***
	γ peaks/θ cycle	4.3 ± 0.1	4.6 ± 0.1	S	0.1078
γ_{ENV} power (V^2)		$9.2 \pm 0.12 \times 10^{-11}$	$5 \pm 0.22 \times 10^{-11}$	M	0.0130*
γ_{ENV}peak frequency (Hz)		7.3 ± 0.3	6 ± 0.2	M	0.0034**
XC(θ- γ_{ENV}) peak		0.29 ± 0.03	0.26 ± 0.02	S	0.2316
XC(θ- γ_{ENV}) peak lag (ms)		-34.8 ± 6.1	-31.4 ± 1.7	S	0.1091
		$p75^{-/-}$			
		Control (n=31)	*Aβ (n=21)*		*p-value*
γ power (V^2)		$8.6 \pm 0.13 \times 10^{-09}$	$8.7 \pm 1.6 \times 10^{-09}$	S	0.4682
γ peak frequency (Hz)		28.7 ± 0.6	33.1 ± 0.9	S	<0.0001****
γ Coefficient of Rhythmicity		0.91 ± 0.01	0.89 ± 0.02	S	0.1481
θ power (V^2)		$3.2 \pm 0.31 \times 10^{-12}$	$2.6 \pm 0.31 \times 10^{-12}$	M	0.1359
θ peak frequency (Hz)		8.9 ± 0.1	8.7 ± 0.2	M	0.1735
θ Coefficient of Rhythmicity		0.7 ± 0.01	0.7 ± 0.01	M	0.4982
γ-θ phase locking	Vector length	$8.7 \pm 0.67 \times 10^{-02}$	$7.3 \pm 0.86 \times 10^{-02}$	S	0.1081
	Phase angle	6 ± 0.04	6.11 ± 0.02	M	0.0664
	γ peaks/θ cycle	4 ± 0.1	4.5 ± 0.1	S	<0.0001****
γ_{ENV} power (V^2)		$1.3 \pm 0.15 \times 10^{-10}$	$1.3 \pm 0.18 \times 10^{-10}$	M	0.4724
γ_{ENV}peak frequency (Hz)		7.2 ± 0.3	6.8 ± 0.3	M	0.3058
XC(θ- γ_{ENV}) peak		0.36 ± 0.02	0.34 ± 0.03	S	0.6077
XC(θ- γ_{ENV}) peak lag (ms)		-28.8 ± 4	-28.7 ± 1.8	S	0.4863
